# Rivaroxaban, a factor Xa inhibitor, improves neovascularization in the ischemic hindlimb of streptozotocin-induced diabetic mice

**DOI:** 10.1186/s12933-015-0243-y

**Published:** 2015-06-16

**Authors:** Tao-Cheng Wu, Jenq-Shyong Chan, Chiu-Yang Lee, Hsin-Bang Leu, Po-Hsun Huang, Jia-Shiong Chen, Shing-Jong Lin, Jaw-Wen Chen

**Affiliations:** Division of Cardiology, Department of Medicine, Taipei Veterans General Hospital, Taipei, Taiwan; Cardiovascular Research Center, National Yang-Ming University, Taipei, Taiwan; Renal Division, Department of Internal Medicine, Taoyuan Armed Forces General Hospital, Taoyuan County, Taiwan; Division of Cardiovascular Surgery, Department of Surgery, Healthcare and Management Center, Taipei Veterans General Hospital, Taipei, Taiwan; Institute of Clinical Medicine, National Yang-Ming University, Taipei, Taiwan; Department of Medical Research and Education, Taipei Veterans General Hospital, No. 201, Section 2, Shih-Pai Road, Taipei 112, Taiwan ROC; Institute of Pharmacology, National Yang-Ming University, Taipei, Taiwan

**Keywords:** Factor Xa inhibitor, Diabetes, Angiogenesis, Endothelial progenitor cells

## Abstract

**Background:**

Factor Xa inhibitor is used for preventing venous thromboembolism (VTE) in adult patients receiving orthopedic operation. However, the role of factor Xa inhibitor, rivaroxaban, in angiogenesis is still unknown.

**Methods and results:**

Streptozotocin (STZ)–induced diabetic mice with model of hind-limb ischemia, were divided into non-diabetic control, diabetic control, and low- and high-dose rivaroxaban treatment groups, in order to evaluate the effect of rivaroxaban in angiogenesis. Doppler perfusion imaging showed that blood flow recovery was significantly increased, and more capillary density occurred in the rivaroxaban treatment group. In vitro studies, human endothelial progenitor cells (EPCs) treated with rivaroxaban had significant functional improvement in migration and senescence under hyperglycemic conditions. Rivaroxaban also increased endothelial nitric oxide synthase (eNOS) as well as vascular endothelial growth factor (VEGF) expressions in hyperglycemia-stimulated EPCs.

**Conclusions:**

Rivaroxaban promoted vessel formation in diabetic mice and improved endothelial progenitor cell function under hyperglycemic conditions. These effects may be associated with enhancement of expression of eNOS and VEGF.

## Background

Diabetes mellitus is a chronic metabolic disease associated with microvascular and macrovascular complications [1–3]. Patients with diabetes have impaired collateral vessel formation in various vascular beds [4]. Recent evidence suggests that bone marrow–derived endothelial progenitor cells (EPCs) play important roles in the process of angiogenesis in response to ischemic conditions [5]; however, patients with diabetes and/or cardiovascular risk factors have a decreased number and function of EPCs [6]. These findings serve as an impetus for the therapeutic targets for high glucose-related vascular complications in diabetic patients.

Rivaroxaban is a direct factor Xa inhibitor that prevents venous thromboembolism and reduces the risk of stroke in atrial fibrillation patients. Low-dose rivaroxaban with aspirin has been approved for use in acute coronary syndrome patients [7]. Rivaroxaban attenuates the leukocyte-platelet-endothelial interaction, which leads to the attenuation of microthrombus formation in diabetic animals [8]. Rivaroxaban has also been shown to inhibit inflammatory mediators and promote lesion stability in atherosclerosis animal studies [9]; however, little is known about the effect of rivaroxaban on angiogenesis in diabetes.

Therefore, we determined whether or not rivaroxaban enhances neovascularization in diabetic mice ischemic tissues and improves EPC function under hyperglycemic conditions.

## Materials and methods

### Ethics statement

Peripheral blood samples were obtained from healthy young adult volunteers. The clinical protocol conform the declaration of Helsinki; the “Institutional Review Board of Taipei Veterans General Hospital” approved the study by expedited review and the subjects provided signed informed consent.

All animal experimental procedures were approved by the Institutional Animal Care Committee of the Taipei Veterans General Hospital (approval reference number IACUC 2011–209). The animal procedures were performed conform the NIH guidelines (Guide for the care and use of laboratory animals) or the guidelines from Directive 2010/63/EU of the European Parliament on the protection of animals used for scientific purposes. Animal sacrifice was performed by CO_2_ asphyxiation followed by cervical dislocation, and all efforts were made to ameliorate animal suffering.

### Animals

Eight-week old mice were purchased from the BioLASCO Taiwan Co., Ltd (FVB mice). Experimental diabetes was induced by daily intra-peritoneal injection of streptozotocin (STZ; 40 mg/kg for FVB mice) in citrate buffer for 5 days and checked plasma sugar was higher than 250 mg/dl to make sure the mice in diabetes (DM group), as described previously in a type I diabetes mellitus model.

### Ischemic hind limb model

The mice without STZ-treated were as non-diabetic control group. STZ-induced diabetic mice were administered with vehicle (0.5 % carboxymethyl cellulose without rivaroxaban), low-dose of rivaroxaban (provided from Bayer, Germany) (0.5 % carboxymethyl cellulose with rivaroxaban 1 mg/kg/day) or high-dose of rivaroxaban (0.5 % carboxymethyl cellulose with rivaroxaban 3 mg/kg/day) daily by gavage [10]. After 2-week treatment, unilateral hind limb ischemia was induced by left femoral artery ligation. The blood flow of hind limb was measured with a Laser Doppler perfusion imager system (Moor Instruments Limited, Devon, UK) before, after surgery, and then weekly. The results were expressed as the ratio of perfusion in the ischemic versus non-ischemic limb [11].

### Evaluation of angiogenesis in the ischemic limb

The mice were sacrificed in 5 weeks after surgery and the limbs were fixed overnight in methanol. The ischemic muscles were embedded in paraffin, and then were deparaffinized in order to incubate with rat monoclonal antibody against murine CD31 (BD PharMingen, San Diego, CA, USA) [10]. New capillaries were identified based on morphology and positive staining for CD31 by using the avidin-biotin-complex technique and Vector Red Chromogenic substrate (Vector Laboratories, Burlingame, CA, USA) after counterstaining with hematoxylin [10]. The visible capillaries were counted under 10 randomly fields. The capillary density was expressed as the number of capillaries/mm^2^ [12].

### Measurement of EPCs mobilization

EPC mobilization was performed with a Calibur flow cytometer (Becton-Dickinson, San Jose, CA, USA), fluorescence-activated cell sorting (FACS). Briefly, peripheral blood was incubated with fluorescein isothiocyanate (FITC) anti-mouse Sca-1 (eBioscience, San Diego, CA, USA) and phycoerythrin (PE) anti-mouse Flk-1 antibodies (VEFGR-2; eBioscience). Isotype-identical antibodies served as controls (Becton-Dickinson, Franklin Lakes, NJ, USA). Circulating EPCs were double-positive gated for Sca-1 and Flk-1 [13], each analysis included 100,000 events. In cell study, hyperglycemia condition was around 25 mM glucose (glucose 20 mM + medium 5 mM).

### Scratch injury model in EPC

Human EPC isolation, cultivation, and characterization were performed as previously described [10, 14–16]. The EPC migration was evaluated by a scratch injury model; confluence EPCs were treated with rivaroxaban (active power provided by Bayer, Germany) for 24 h and incubated under hyperglycemic conditions for 4 days. After serum-starvation of EPCs overnight, and a scratch injury was applied with a scalpel. Then, EPCs sprouting was examined before and 12/24 h after scratching [10].

### Measurement of tube formation assay

EPC tube formation assay was analyzed with the In Vitro Angiogenesis assay kit (Chemicon, USA). EPCs were placed onto a matrix with medium for 16 h. Tubule formation was inspected under inverted light microscopy. Six random fields were used to calculate the average of complete tubes formed by cells using Image-Pro Plus software (USA) [10].

### Measurement of EPC senescence assay

Cellular aging was analyzed with senescence cell staining kit (Sigma, USA). EPCs were fixed (2 % formaldehyde and 0.2 % glutaraldehyde), then incubated with fresh X-gal staining solution (1 mg/mL X-gal, 5 mM potassium ferricyanide, and 2 mM MgCl_2_) in the absence of CO_2_. Green-stained cells were counted and the percentage of β-galactosidase-positive cells was calculated [12], and senescent cells were expressed as % of the total cell number.

### Western blotting analysis

The proteins were probed with monoclonal antibodies against eNOS, phosphorylated eNOS (p-eNOS), VEGF, and actin (Chemicon, USA). Densitometric analysis was conducted with ImageQuant (Promegam, USA) software [12].

### Statistical analysis

Data are expressed as mean ± standard error of mean (SEM). Statistical analysis was performed with unpaired Student’s *t*-test or analysis of variance, followed by Scheffe’s multiple comparison *post hoc* test using Statistical Package of the Social Sciences software (version 14; SPSS, Inc., Chicago, IL, USA). *P* value < 0.05 was considered statistically significant [10].

## Results

### Rivaroxaban enhances blood flow recovery in diabetic mice with hind limb ischemia

**Fig. 1:**
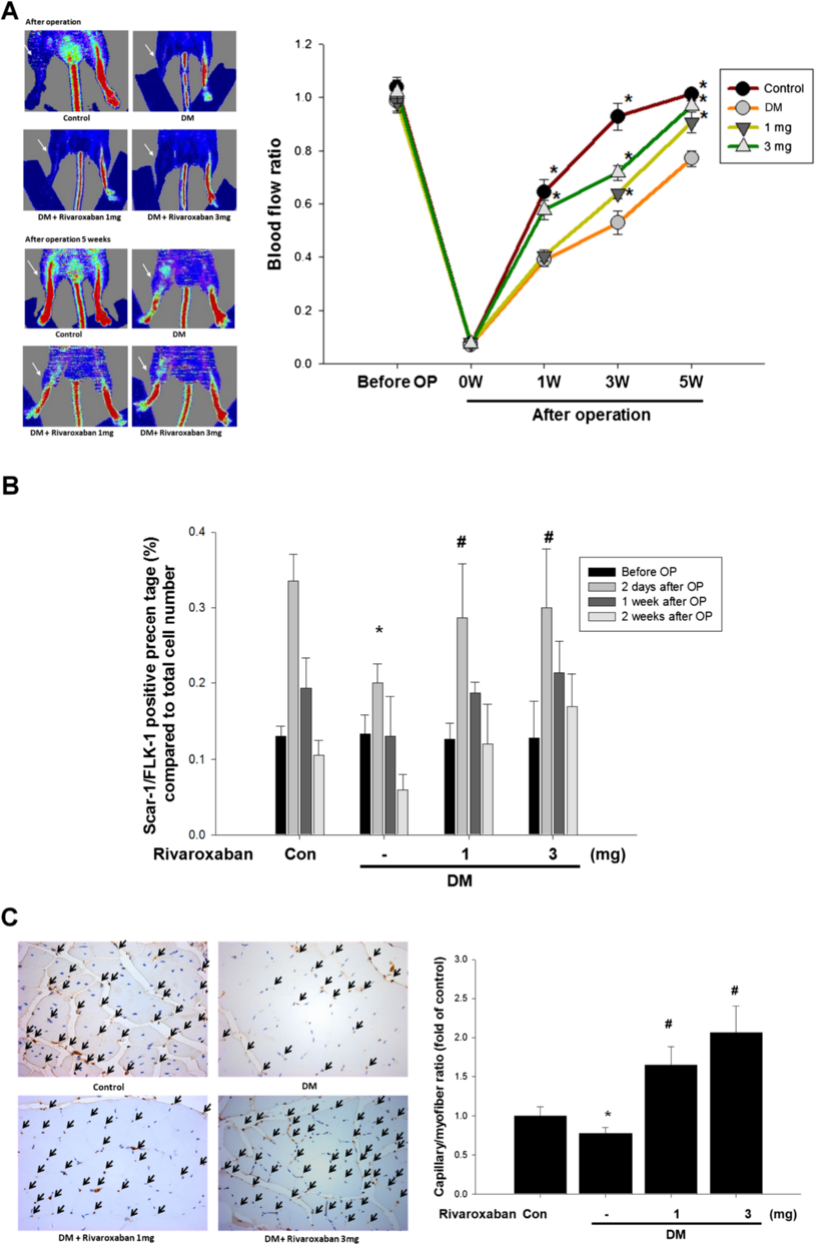
Effects of rivaroxaban on blood flow recovery and new vessel formation in STZ-induced diabetic mice after hind limb ischemia. **a** Representative results of laser Doppler measurements pre-operatively and 5 weeks after hind limb ischemia surgery in non-diabetic control mice, diabetic control mice, diabetic mice treatment with low-dose rivaroxaban, and diabetic mice with high-dose rivaroxaban. Color scale illustrates blood flow variation from minimal (*dark blue*) to maximal (*red*) values. *Arrows* indicate ischemic limb after hind limb ischemia surgery. **b** Endothelial progenitor cell (EPC; defined as Sca-1^+^/Flk-1^+^ cells) mobilization after tissue ischemia was determined by flow cytometry in mice. (*P < 0.05 compared with baseline; *n* = 6). **c** Mice were sacrificed 5 weeks after surgery and capillaries in the ischemic muscles were visualized by anti-CD31 immunostaining. Results are the mean ± standard error of mean (SEM). (*P < 0.05 compared with DM control; *n* = 6)

To evaluate the angiogenic effect of rivaroxaban, unilateral hind limb ischemia surgery was performed in non-diabetic and STZ-induced diabetic mice. Compared to non-diabetic mice, the STZ-induced diabetic mice had delayed blood flow recovery after surgery (Fig. 1a); however, administration of low- or high-dose rivaroxaban significantly enhanced flow recovery in diabetic mice. Anti-CD31 immunostaining showed decreased capillary density in diabetic mice compared to control mice, but treatment with rivaroxaban significantly increased capillary density in muscles in diabetic mice (Fig. 1b). Rivaroxaban also increased EPC proliferation after surgery in diabetic mice (Fig. 1c).

### Rivaroxaban increased VEGF and eNOS expression in diabetic mice with hind limb ischemia

**Fig. 2:**
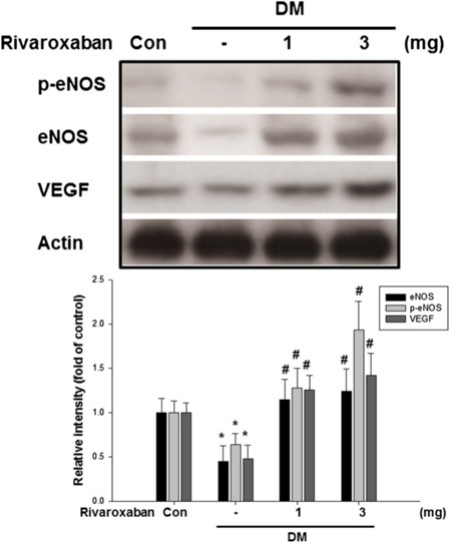
Effects of rivaroxaban on eNOS and VEGF production in ischemic tissues of mice. Impaired expression of eNOS, p-eNOS, and VEGF of ischemic tissues in diabetic control mice compared to non-diabetic control mice. Treatment with rivaroxaban in diabetic mice promoted eNOS, p-eNOS, and VEGF production of ischemic tissues compared to diabetic control mice. The expression of VEGF was enhanced in a dose-dependent effect of diabetic mice treated with rivaroxaban. Each *bar graph* shows the summarized data from six separate experiments by densitometry after normalization. Data are the mean ± SEM. (*P < 0.05 compared with non-diabetic control mice, ^#^ *P* < 0.05 compared with diabetic control mice; *n* = 6)

Impaired expression of eNOS and VEGF in the ischemic muscular tissues of diabetic mice was noted compared to the non-diabetic mice. Rivaroxaban significantly increased eNOS and VEGF expressions in the ischemic muscular tissues in diabetic mice (Fig. 2). These results suggested that rivaroxaban may enhance eNOS and VEGF production in diabetes.

### Rivaroxaban improves hyperglycemia-suppressed EPC mobilization, tube formation, and senescence in vitro

**Fig. 3:**
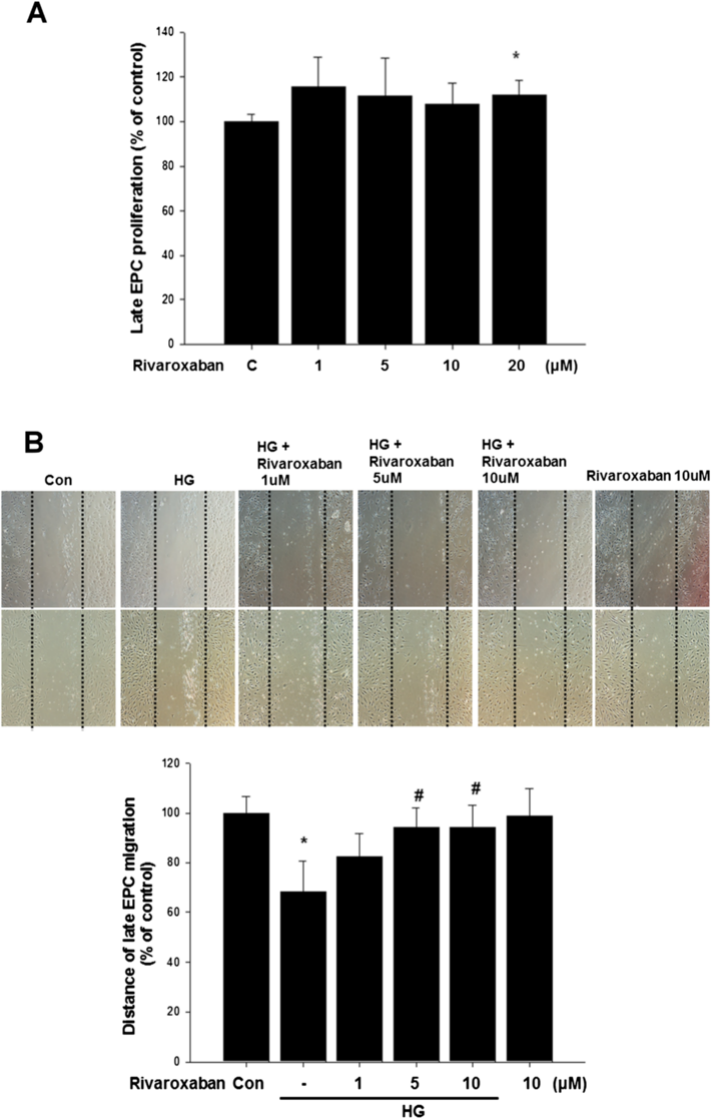
Effects of rivaroxaban on human EPC migration in vitro. **a** The cell viability assays evaluated the toxicity of rivaroxaban in late EPC proliferation. **b** The migratory function of EPCs was evaluated using a scratch injury model. Data are the mean ± SEM. (*P < 0.05 compared with control, ^#^ *P* < 0.05 compared with hyperglycemic conditions, *n* = 4 for each experiment)

To evaluate the effect of rivaroxaban on EPCs, the scratch test for migratory function of EPCs was performed. Compared with the control group, EPCs under hyperglycemic conditions significantly decreased EPC migration (Fig. 3); however, treatment with rivaroxaban significantly improved hyperglycemia-suppressed late EPC migratory function (Fig. 3b).

**Fig. 4:**
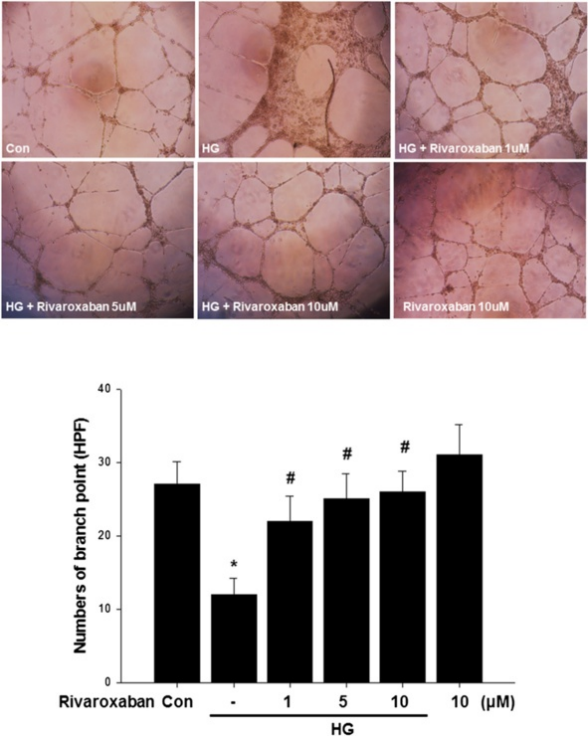
Effects of rivaroxaban on human EPC tube formation in vitro. An in vitro angiogenesis assay for EPCs used ECMatrix gel. Cells were stained with crystal violet, and the averages of the total area of complete tubes formed by cells were compared using computer software. Data are the mean ± SEM. (*P < 0.05 compared with control, ^#^ *P* < 0.05 compared with HG group, *n* = 4 for each experiment)

The tube formation of EPCs was also investigated; the capillary density was significantly reduced in hyperglycemic conditions. Treatment of EPCs with rivaroxaban significantly increased tube formation of EPCs compared with the drug treatment group in hyperglycemic conditions (Fig. 4).

**Fig. 5:**
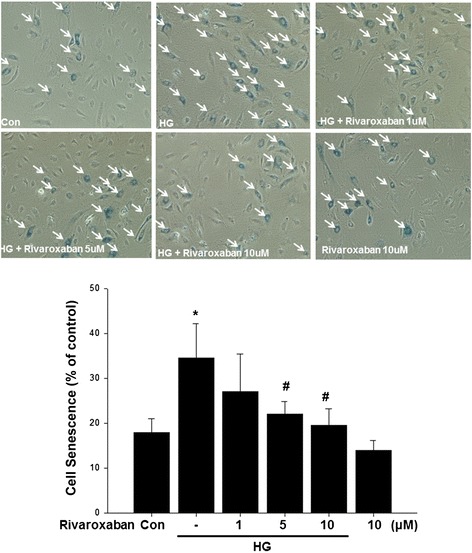
Effects of rivaroxaban on human EPC senescence in vitro. To determine the onset of cellular aging, acid β-galactosidase was used as a biochemical marker for acidification (typical for EPC senescence). Data are the mean ± SEM. (*P < 0.05 compared with control, ^#^ *P* < 0.05 compared with HG group, *n* = 4 for each experiment)

Compared with the control, hyperglycemic condition significantly increased senescence-associated β-galactosidase-positive EPCs. Treatment of EPCs with rivaroxaban significantly reduced the percentage of senescence EPCs in hyperglycemic conditions (Fig. 5).

### Rivaroxaban up-regulates eNOS phosphorylation and enhances VEGF expression in hyperglycemic conditions

**Fig. 6:**
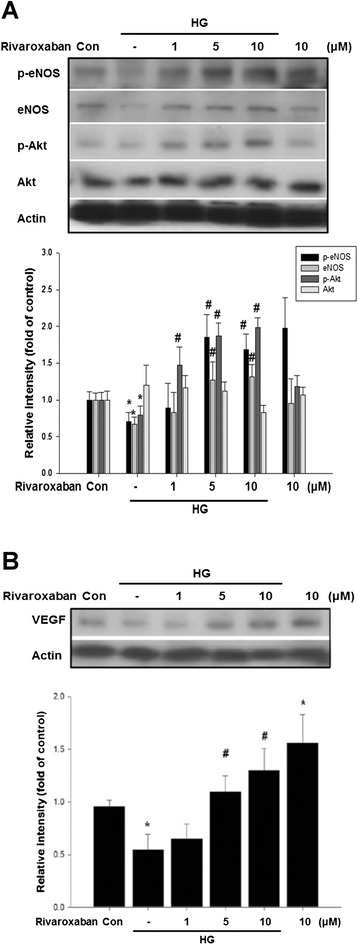
Effects of rivaroxaban on eNOS, p-eNOS, and VEGF production in cultured human EPCs. **a** Administration of rivaroxaban (1, 5, and 10 μM) for 24 h, followed by treatment of EPCs under hyperglycemic conditions. Rivaroxaban up-regulated hyperglycemic impaired e-NOS phosphorylation, eNOS, and Akt phosphorylation. **b** In addition, administration of rivaroxaban also promoted VEGF production under hyperglycemic conditions. Each *bar graph* shows the summarized data from four separate experiments by densitometry after normalization. Data are the mean ± SEM. (*P < 0.05 compared with control, ^#^ *P* < 0.05 compared with HG group, *n* = 4 for each experiment)

To understand the effect of rivaroxaban on eNOS activation in EPCs, treatment of EPCs with low- and high-dose rivaroxaban in hyperglycemic conditions significantly up-regulated hyperglycemic-impaired eNOS production. In addition, rivaroxaban also up-regulated Akt action and promoted VEGF production in hyperglycemic conditions. These results suggested that rivaroxaban may enhance eNOS, Akt, and VEGF production in EPCs after hyperglycemic stimulation (Fig. 6).

## Discussion

This is the first study to report the role of rivaroxaban in angiogenesis in diabetes. Specifically, we demonstrated that rivaroxaban enhanced blood flow recovery in response to tissue ischemia in diabetic mice. The eNOS and VEGF expression were increased in ischemic tissues in diabetic mice after rivaroxaban treatment. Rivaroxaban improved EPC functions, such as migration, tube formation, and senescence. In addition, rivaroxaban enhanced eNOS and VEGF activities of EPCs under hyperglycemic conditions. Our findings suggest that rivaroxaban has beneficial effects on EPCs under hyperglycemic conditions and might provide vascular protection in clinical settings.

EPCs are known as mediators of endothelial repair. Insulin resistance, including nitric oxide (NO) bioavailability, and production of reactive oxidative stress (ROS) potentially interferes with EPC function and decreased levels of circulating EPCs were noted in diabetes [17–20]; however, there is impaired angiogenesis in the peripheral vasculature (not only decreased endothelial cell proliferation, but also reduced growth factor in diabetic patients) [21]. Under hyperglycemic conditions, down-regulation of VEGF causes impairment of angiogenesis, EPC dysfunction, and defective lymphatic vascular formation [22, 23]. But Yu et al. found the function of ex vivo expanded EPCs from autologous bone marrow transplantation without damage regardless of glycemic state [24]. Recent studies indicate that commonly used drugs in diabetes, including ACE inhibitors, DPP4 inhibitors, GLP-1 agonists, insulin, metformin, statins, or thiazolidinediones, may increase the number of EPCs and improve EPC function [25]. For the first time, we have demonstrated that the factor Xa inhibitor, rivaroxaban, enhances VEGF and eNOS expression of EPCs and improves EPC function, including migration, tube formation, and senescence, in experimental diabetes. Thus, rivaroxaban might increase angiogenesis under hyperglycemic conditions.

Factor Xa plays a role in coagulation for the intrinsic and extrinsic pathways, and along with thrombin [26], mediates signal transduction through the activation of protease-activated receptors (PARs) [27]. Factor Xa has been shown to act as a mitogenic agent [26] in vascular smooth muscle cells [28] and cytokine production in endothelial cells [29]. Lange et al. showed that PAR-1 mediates the anti-angiogenic effect of factor Xa [26]. Yavuz et al. reported a dose dependent anti-angiogenic behavior of Rivaroxaban and only anti-angiogenic effect was shown in high toxic doses [30]. Here, we demonstrated that rivaroxaban possesses pro-angiogenic properties in hind limb ischemia in diabetic mice and through the increased secretion of growth factor improves angiogenesis in diabetes.

Rivaroxaban may reduce venous thromboembolism rates in total hip or knee arthroplasty patients [31]; however, some evidence shows that rivaroxaban has some non-hemostatic functions. Zhou et al. reported that rivaroxaban stabilizes atherosclerotic plaques in a mouse model [9]. Low-dose rivaroxaban reduces the risk of death from stroke, cardiovascular causes, or myocardial infarction, in acute coronary syndrome patients [32]. Ishibashi et al. found rivaroxaban might block the interaction between advanced glycation end products (AGEs)-RAGE axis and coagulation system to prevent thromboembolic complications in diabetes [33].

Hollborn and colleagues found that the activated blood coagulation factor Xa induced chemotaxis of retinal pigment epithelial cells and stimulated the release of angiogenic growth factors such as VEGF [34]. The inhibition of factor Xa might suppress coagulation-induced angiogenesis. It might suggest that rivaroxaban might have a direct effect on the secretion of VEGF from the EPCs in this study. In this study, rivaroxaban enhanced blood flow recovery and new vessel formation in experimental diabetes. In in vitro assays, incubation of cultured EPCs with rivaroxaban up-regulated eNOS phosphorylation and improved EPC functions. These findings suggest a vasoprotective effect of rivaroxaban under hyperglycemic conditions via the NO pathways.

In our study, rivaroxaban seems to have direct effects on endothelial progenitor cell to secret VEGF in vitro and promote neovascularization in diabetic mice in vivo. Although the best way to evaluate the angiogenic effects of rivaroxaban should use factor Xa knock-out animal models, our results still suggest the role of rivaroxaban in angiogenesis in diabetes. The molecular mechanisms of angiogenic factors of rivaroxaban in angiogenesis should be further evaluated. We are also interested in evaluating the correlation between the EPCs function and ABI value in diabetes, non-diabetes and healthy groups in our near future human study.

## Conclusion

The present study showed that rivaroxaban improves blood flow recovery and increases neovascularization in diabetic mice with hind limb ischemia. Rivaroxaban also promotes the functions of EPCs, including migration, tube formation, and senescence, and via NO-related pathways. These findings demonstrate that rivaroxaban may also play a therapeutic role in patients with diabetic foot or peripheral artery disease.

## Abbreviations

eNOS: Endothelial nitric oxide synthase
EPCs: Endothelial progenitor cells
FACS: Fluorescence-activated cell sorting
FITC: Fluorescein isothiocyanate
IACUC: Institutional Animal Care Committee
NO: Nitric oxide
PE: Phycoerythrin
ROS: Reactive oxidative stress
SEM: Standard error of mean
STZ: Streptozotocin
VEGF: Vascular endothelial growth factor
VTE: Venous thromboembolism

## References

[1] Wilke T, Mueller S, Groth A, Fuchs A, Seitz L, Kienhofer J (2015). Treatment-dependent and treatment-independent risk factors associated with the risk of diabetes-related events: a retrospective analysis based on 229,042 patients with type 2 diabetes mellitus. Cardiovasc Diabetol.

[2] Blonde L, Pencek R, MacConell L (2015). Association among weight change, glycemic control, and markers of cardiovascular risk with exenatide once weekly: a pooled analysis of patients with type 2 diabetes. Cardiovasc Diabetol.

[3] Vallejo S, Palacios E, Romacho T, Villalobos L, Peiro C, Sanchez-Ferrer CF (2014). The interleukin-1 receptor antagonist anakinra improves endothelial dysfunction in streptozotocin-induced diabetic rats. Cardiovasc Diabetol.

[4] Rivard A, Silver M, Chen D, Kearney M, Magner M, Annex B (1999). Rescue of diabetes-related impairment of angiogenesis by intramuscular gene therapy with adeno-VEGF. Am J Pathol.

[5] Gallagher KA, Liu ZJ, Xiao M, Chen H, Goldstein LJ, Buerk DG (2007). Diabetic impairments in NO-mediated endothelial progenitor cell mobilization and homing are reversed by hyperoxia and SDF-1 alpha. J Clin Invest.

[6] Lee PS, Poh KK (2014). Endothelial progenitor cells in cardiovascular diseases. World J Stem Cells.

[7] Plosker GL (2014). Rivaroxaban: a review of its use in acute coronary syndromes. Drugs.

[8] Iba T, Aihara K, Yamada A, Nagayama M, Tabe Y, Ohsaka A (2014). Rivaroxaban attenuates leukocyte adhesion in the microvasculature and thrombus formation in an experimental mouse model of type 2 diabetes mellitus. Thromb Res.

[9] Zhou Q, Bea F, Preusch M, Wang H, Isermann B, Shahzad K (2011). Evaluation of plaque stability of advanced atherosclerotic lesions in apo E-deficient mice after treatment with the oral factor Xa inhibitor rivaroxaban. Mediators Inflamm.

[10] Huang PH, Lin CP, Wang CH, Chiang CH, Tsai HY, Chen JS (2012). Niacin improves ischemia-induced neovascularization in diabetic mice by enhancement of endothelial progenitor cell functions independent of changes in plasma lipids. Angiogenesis.

[11] Lee AW, Chen TL, Shih CM, Huang CY, Tsao NW, Chang NC (2010). Ursolic acid induces allograft inflammatory factor-1 expression via a nitric oxide-related mechanism and increases neovascularization. J Agric Food Chem.

[12] Huang PH, Tsai HY, Wang CH, Chen YH, Chen JS, Lin FY (2010). Moderate intake of red wine improves ischemia-induced neovascularization in diabetic mice–roles of endothelial progenitor cells and nitric oxide. Atherosclerosis.

[13] Huang PH, Chen JW, Lin CP, Chen YH, Wang CH, Leu HB (2012). Far infra-red therapy promotes ischemia-induced angiogenesis in diabetic mice and restores high glucose-suppressed endothelial progenitor cell functions. Cardiovasc Diabetol.

[14] Huang PH, Chen YH, Wang CH, Chen JS, Tsai HY, Lin FY (2009). Matrix metalloproteinase-9 is essential for ischemia-induced neovascularization by modulating bone marrow-derived endothelial progenitor cells. Arterioscler Thromb Vasc Biol.

[15] Huang PH, Chen YH, Tsai HY, Chen JS, Wu TC, Lin FY (2010). Intake of red wine increases the number and functional capacity of circulating endothelial progenitor cells by enhancing nitric oxide bioavailability. Arterioscler Thromb Vasc Biol.

[16] Huang PH, Huang SS, Chen YH, Lin CP, Chiang KH, Chen JS (2010). Increased circulating CD31+/annexin V+ apoptotic microparticles and decreased circulating endothelial progenitor cell levels in hypertensive patients with microalbuminuria. J Hypertens.

[17] Fadini GP, Miorin M, Facco M, Bonamico S, Baesso I, Grego F (2005). Circulating endothelial progenitor cells are reduced in peripheral vascular complications of type 2 diabetes mellitus. J Am Coll Cardiol.

[18] Yan J, Tie G, Park B, Yan Y, Nowicki PT, Messina LM (2009). Recovery from hind limb ischemia is less effective in type 2 than in type 1 diabetic mice: roles of endothelial nitric oxide synthase and endothelial proggenitor cells. J Vasc Surg.

[19] Olikonomou D, Kopf S, von Bauer R, Djuric Z, Cebola R, Sander A (2014). Influence of insulin and glargine on outgrowth and number of circulating endothelial progenitor cells in type 2 diabetes patients: a partially double-blind, randomized, three-arm unicenter study. Cardiovasc Diabetol.

[20] Kuliszewski MA, Ward MR, Kowalewski JW, Smith AH, Stewart DJ, Kutryk MJ (2013). A direct comparison of endothelial progenitor cell dysfunction in rat metabolic syndrome and diabetes. Atherosclerosis.

[21] Martin A, Komada MR, Sane DC (2003). Abnormal angiogenesis in diabetes mellitus. Med Res Rev.

[22] Maruyama K, Asai J, Ii M, Thorne T, Losordo DW, D’Amore PA (2007). Decreased macrophage number and activation lead to reduced lymphatic vessel formation and contribute to impaired diabetic wound healing. Am J Pathol.

[23] Verheul HM, Pinedo HM (2007). Possible molecular mechanisms involved in the toxicity of angiogenesis inhibition. Nat Rev Cancer.

[24] Yu P, Li Q, Liu Y, Zhang J, Seldeen K, Pang M (2015). Pro-angiogenic efficacy of transplanting endothelial progenitor cells for treating hindlimb ischemia in hyperglycemic rabbits. J Diabetes Complications.

[25] Desouza CV (2013). Does drug therapy reverse endothelial progenitor cell dysfunction in diabetes?. J Diabetes Complications.

[26] Lange S, Gonzalez I, Pinto MP, Arce M, Valenzuela R, Aranda E (2014). Independent anti-angiogenic capacities of coagulation factors X and Xa. J Cell Physiol.

[27] Rao LV, Pendurthi UR (2005). Tissue factor-factor VIIa signaling. Arterioscler Thromb Vasc Biol.

[28] Herbert J, Bono F, Herault J, Avril C, Dol F, Mares A (1998). Effector protease receptor 1 mediates the mitogenic activity of factor Xa for vascular smooth muscle cells in vitro and in vivo. J Clin Invest.

[29] Senden NH, Jeunhomme TM, Heemskerk JW, Wagenvoord R, van’t Veer C, Hemker HC (1998). Factor Xa induces cytokine production and expression of adhesion molecules by human umbilical vein endothelial cells. J Immunol.

[30] Yavuz C, Caliskan A, Karahan O, Yazici S, Guclu O, Demirtas S (2014). Investigation of the antiangiogenic behaviors of rivaroxaban and low molecular weight heparins. Blood Coagul Fibrinolysis.

[31] Borris LC (2010). Rivaroxaban and dabigatran etexilate: two new oral anticoagulants for extended postoperative prevention of venous thromboembolism after elective total hip arthroplasty. Arch Orthop Trauma Surg.

[32] Mega JL, Braunwald E, Wiviott SD, Bassand JP, Bhatt DL, Bode C (2012). Rivaroxaban in patients with a recent acute coronary syndrome. N Engl J Med.

[33] Ishibashi Y, Matsui T, Ueda S, Fukami K, Yamagishi S (2014). Advanced glycation end products potentiate citrated plasma-evoked oxidative and inflammatory reactions in endothelial cells by up-regulating protease-activated receptor-1 expression. Cardiovasc Diabetol.

[34] Hollborn M, Kohen L, Werschnik C, Tietz L, Wiedemann P, Bringmann A (2012). Activated blood coagulation factor X (FXa) induces angiogenic growth factor expression in human retinal pigment epithelial cells. Invest Ophthalmol Vis Sci.

